# Triptolide inhibits the progression of Glioblastoma U251 cells *via* targeting PROX1

**DOI:** 10.3389/fonc.2023.1077640

**Published:** 2023-03-10

**Authors:** Chao Yuan, Yanli Liao, Shengjie Liao, Mi Huang, Duanzhuo Li, Weibin Wu, Yi Quan, Liqiang Li, Xin Yu, Wenxia Si

**Affiliations:** ^1^ Department of Scientific Research and Experiment Center, Zhaoqing Medical College, Zhaoqing, Guangdong, China; ^2^ Department of Oncology, Zhaoqing First People’s Hospital Affiliated to Zhaoqing Medical College, Zhaoqing, Guangdong, China

**Keywords:** triptolide (PubChem CID: 107985), Glioblastoma multiforme, PROX1, apoptosis, migration and invasion, proliferation

## Abstract

**Background:**

Glioblastoma multiforme (GBM) is the most lethal brain cancer in adults, characterized by rapid growth, extensive invasiveness, and poor prognosis, and there is still a lack of effective treatments. Here, we aimed to explore the role of triptolide (TPL), purified from *Tripterygium wilfordii* Hook F, on glioblastoma cell growth, apoptosis, proliferation, migration and invasion, as well as potential underlying mechanisms.

**Methods:**

The publicly available clinical data of Brain Lower Grade Glioma (LGG) from The Cancer Genome Atlas (TCGA) had been screened to observe PROX1 expression. The Kaplan-Meier analysis was used to analyze the relationship between PROX1 expression and GBM prognosis. CCK8, cell cycle, EDU, apoptosis, wound healing, and transwell assays were performed to detect the effects of TPL on glioblastoma U251 cell viability, cell cycle, proliferation, apoptosis, migration and invasion, respectively. Further, a soft agar colony assay was used to calculate the growth of glioblastoma cells. The qRT-PCR and western blot were conducted to quantify PROX1 mRNA and protein levels. The transcriptional regulation of TPL was detected by Dual luciferase reporter assay.

**Results:**

We found that TPL inhibited glioblastoma cell viability, proliferation, cell cycle, migration and invasion, but enhanced apoptosis in a dose-dependent manner. The expression of cell cycle inhibitor, P21, and pro-apoptosis factor, Bax was increased, while invasion-related factors MMP2 and MMP9 were silenced after TPL treatments. Mechanistically, TPL showed transcriptional inhibition of PROX1 appearance. Moreover, ectopic expression of PROX1 partially rescued the effects of TPL on glioblastoma cell viability, proliferation, apoptosis, migration and invasion, and on the expression of cell function-related genes.

**Conclusion:**

This study verified that TPL inhibited the progression of glioblastoma cells by transcriptionally depressing the expression of PROX1.

## Introduction

Glioblastoma (GBM) is the most common primary central nervous system malignancy in adults, and patients have a poor survival period of about 15 months (1). Currently, the main treatment strategies for GBM are surgical resection followed by radiotherapy and temozolomide (TMZ) chemotherapy (2). However, due to the infiltrative nature of GBM, Traditional surgical methods cannot completely remove invasive tumor cells and failed to prevent tumor recurrence. Targeted drugs such as bevacizumab (anti-angiogenic factor), alectinib (ALK gene), and everolimus (mTOR inhibitor), have been successively used in the precise treatment of GBM, and significantly improved progression-free survival (PFS) in GBM patients (3, 4). Immunotherapy has emerged as a potent approach for treating aggressive cancers, such as non–small-cell lung tumors (NSCLC) and melanoma, but does not affect GBM (4). Therefore, it is urgent to develop newer and more effective therapeutic strategies and agents to treat GBM.

Triptolide (TPL), a main active component purified from the Chinese herb *Tripterygium wilfordii* Hook F, has shown antitumor activities in various cancer cell types (5). Numerous studies have proved that TPL is a broad-spectrum tumor inhibitor for various cancers such as leukemia, breast cancer, pancreatic cancer, and lung cancer. TPL was reported to inhibit the cell proliferation and tumorigenesis of neuroblastoma and the NF-κB pathway was involved in the TPL-induced neuroblastoma cell apoptosis (6). In terms of Glioblastoma, Sai et al. demonstrated that TPL inhibits NF-κB signaling and enhances TMZ-induced apoptosis (7). However, there are still few studies on its anti-tumor effect in glioblastoma, and the underlying mechanism remains largely unknown.

The homeobox protein Prospero-related homeobox 1 (PROX1) is a homeobox transcription factor that plays a critical role in the development of organs during embryogenesis (8). Originally, PROX1 served as a specific marker to lymphatic vessels and has an essential role in lymphangiogenesis (9). Outside the lymphatic system, PROX1 is expressed in several tissues including the heart, liver, skeletal muscle, pancreas, kidney, and nervous system (10). In addition, PROX1 has also been found to be involved in the occurrence and development of various tumor types. PROX1 regulates the proliferation and differentiation of tumor cells through different transcriptional pathways, but whether PROX1 plays the role of “tumor suppressor” or “tumor promoter” is still controversial. It has been reported that the high expression of PROX1 is positively associated with the proliferation of colon cancer cells and the metastasis of liver cancer cells (11, 12); however, its high expression has an inhibitory effect on the proliferation of esophageal and pancreatic cancers (13, 14). It is worth mentioning that the expression of PROX1 is different in GBM with different malignant degrees. Tamador Elsir et al. have shown that High-grade gliomas (grades III and IV) have a higher expression of PROX1, and PROX1 can be used as a molecular marker for the diagnosis of astrocytic gliomas (15). Kenney R. et al. also suggest that PROX1 can serve as a new prognostic biomarker for astrocytomas (16). Xu et al. recently reveal that PROX1 is an oncogene in GBM *via* activating the NF-kB signal (17).

In this study, we further validated the anti-tumor effects of TPL and explored the role of PROX1 on TPL-induced inhibition of cell proliferation, migration and invasion, and enhancement of apoptosis using the human glioblastoma U251 cell line. These results will help to understand the critical role of TPL in glioblastoma cell proliferation and apoptosis and provide new therapeutic medicine for glioblastoma therapy.

## Materials and methods

### Cell culture

Human glioblastoma U251 cells were obtained from BeNa Culture Collection (BNCC) (Henan, P.R. China). Cells were cultured in RPMI 1640 Medium (Gibco) with 10% FBS, and maintained in a humidity incubator (Thermo Fisher Scientific, USA) at 37°C with 5% CO2. LipoFectMax™ 3000 Transfection Reagent (ABP Biosciences) was used to transfect the U251 cells.

### Drugs and regents

TPL was purchased from PUSH Bio-technology (Chengdu, P.R. China), dissolved in DMSO to a storage concentration of 10 mM and stored at −20°C. Serum-free DMEM was used to dilute TPL before the experiments. Puromycin aminonucleoside (1.5 μg/ml) was obtained from MedChemExpress.

### qRT-PCR assay

Total RNA was extracted from the cells using the TRIzol kit (Takara, Dalian, China) as described previously (18). 1 μg RNA was used to transcribe cDNA using the HiScript II Q RT SuperMix (R223-01, Vazyme). Quantitative reverse transcription polymerase chain reaction (qRT-PCR) was performed using a qTOWER^3^84G(analytikjena). The results were analyzed using the 2^-ΔΔCt^ method and GAPDH as an internal reference. The primers used in the study were as follows:

PROX1Forward Primer: AAAGGACGGTAGGGACAGCAT, Reverse Primer: CCTTGGGGATTCATGGCACTAA; GAPDH Forward Primer: ATGGGGAAGGTGAAGGTCG, Reverse Primer: GGGGTCATTGATGGCAACAATA.

### Western blot assay

Western blot analysis was carried out as described previously (19). Briefly, U251 cells were cultured and treated as indicated demand. The total protein was extracted using RIPA lysis buffer (Beyotime), and the cell lysates were subjected to western blot. The polyclonal antibodies (pAb) against PROX1, Bax, P21, and GAPDH were from Proteintech. The anti-MMP2 and MMP9 antibodies were from Affinity Biosciences.

### Luciferase assay

The transcription activation activity of TPL was determined using luciferase assays with a PRXO1-Luc reporter, which was cloned as before (20). U251cells were cultured in 24 wells and co-transfected with PROX1-LUC (0.25mg) and pRL-TK plasmid (10ng, internal control). 24 hours later cells suffered to 0, 50, 100, or 200 nM TPL treatments. 24h later, the cells were lysed, and the lysates were used for luciferase assay.

### CCK8 assay

Cell counting kit-8 (CCK-8) assay (Beyotime) was performed to evaluate the U251cell viability as before (18). Briefly, 1x10^5^ U251cells were seeded in a 96-well plate (NEST, China) for 24 h, and then treated with different concentrations of TPL for another 24h. The CCK-8 solution (10 μl) was added to the culture medium of each well. After that, the cell plate was incubated for 4 hours at 37°C, and the absorbance of each well at 450 nm was recorded using a microplate reader (Spark, TECAN).

### Cell cycle assay

The effect of TPL on the cell cycle in glioblastoma was analyzed using the Cell Cycle Staining Kit (Beyotime). And the cell cycle assay was performed as described previously (21). After treatment with TPL for 24h, U251 cells were trypsinized, fixed in 70% ice-cold ethanol for 24 h and washed with PBS, then incubated with 100 mg/mL RNase and 4 mg/mL propidium iodide (PI) in 500 μl PBS. The cell cycle phase was analyzed using a cytoflex (Beckman Coulter).

### Apoptosis assay

The effects of TPL on glioma cell apoptosis were measured using the Annexin V-FITC/PI apoptosis kit (Beyotime). U251 cells were treated with TPL or TPL + puromycin (1.5μg/mL) for 24h. Then the cells were washed, digested with trypsin without EDTA, and collected for staining. 200 μl staining buffer with Annexin V-FITC and PI was added to the cells and incubated at room temperature for 15 min, and analyzed with a cytoflex (Beckman Coulter). We calculated the percentage of apoptotic cells by the percentage of cells in the quadrant of AnnexinV-FITC-positive, PI-positive, or PI-negative.

### Wound healing assay

U251cells (80% confluence) were plated into 12-well culture plates (NEST, China), and cultured overnight. A scratch was created artificially using the tip of a 10 μl pipette gun in the middle of the adherent cells. Finally, the medium with different concentrations of TPL was added to the cells. Phase contrast images were taken at 0, 6 and 24h after scratching using an Olympus light microscope with a 10x objective lens. Then the relative migration cells and width were measured using Image J.

### Transwell migration assay

For migration experiments, U251cells were treated with different concentrations of TPL for 24hr. Then cells were harvested and re-cultured in the matrigel-coated (Biozellen, rehydrated with medium, 1:50) transwell chamber (8mm, Corning) with 200 µL serum-free DMEM, while the lower part filled with 500 µL DMEM containing 10% FBS. After Incubating for 12 hr, the cells which were attached inside of the filter were wiped with a cotton swab, while the invaded cells on the lower surface were fixed with methyl alcohol for 15 min, and stained with 0.1% crystal violet for 20 min. Then PBS was used to wash the cells until washing out the excess dye. The cells were counted under the Olympus microscope at a magnification of 100x and quantified by Image J.

### Proliferation assay

Cell proliferation was monitored using the iClick™ EdU Andy Fluor™ 647 Imaging Kit (ABP Biosciences) following the manufacturer’s instructions. U251 cells were cultured in 24 wells and treated with 0, 50, 100 nM TPL and 100 nM TPL+pcDNA3.1-PROX1 expression plasmid for 24 h, and incubated with 10 μM EdU for 12 h, fixed with 4% paraformaldehyde for 15 min at 37°C, permeabilized in 0.3% TritonX-100 and washed twice with PBS containing 0.3% BSA. Then, 100 μl of Apollo 567 stain reaction buffer was added for 30 min and washed twice with 0.3% BSA. The cells were stained with 100 μl of DAPI (dilution at 1: 2000) for 20 min at room temperature and imaged under the Olympus microscope at a magnification of 400x. The rate of cell proliferation was calculated with the percentage of EdU-positive red cells over DAPI-stained cells (blue) × 100%.

### Soft agar colony formation assay

U251 cells were cultured in 12 wells and treated with 0, 50, and 100 nM TPL, and one well transfected with pcDNA3.1-PROX1 plasmid and 100 nM TPL and harvested as single-cell suspension. We resuspended 1 × 10^4^ cells in 500 μl of a 1:1 mix of medium and Matri-gel (Biozellen), and 100 μl drops were placed on precooled 24-well plates and allowed to solidify for 5 minutes at 4°C. Another 1ml of fixing solution was added for 15 minutes, and then 1ml of the medium was added and cultured in an incubator for 1-2 weeks. Plates were stained with crystal violet (Beyotime) and washed to remove excess crystal violet. The number of colonies was scored with Image J.

### Statistical analysis

All experiments were repeated independently at least three times and GraphPad Prism 6.0 software was used for statistical analyses. All data were presented as mean ± SD. The Student’s t-test and one-way ANOVA were employed to compare differences between groups. *p <*0.05 was considered to be statistically significant.

## Results

### PROX1 expression is associated with poor prognosis in glioblastoma patients

**Figure 1:**
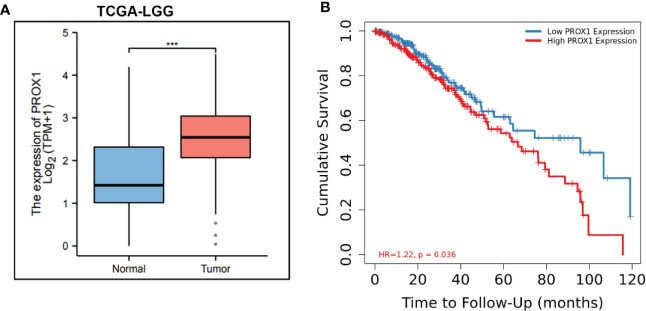
PROX1 expression is associated with unfavorable prognosis in LGG Patients. **(A)** The expression of PROX1 mRNA in lower grade glioma (LGG) from TCGA database. Among them, 516 tumor and 768 non-tumor samples were used for analysis, ***, *p*<0.001. **(B)** Survival curve (Kaplan-Meier) of LGG patients with relative high and low expression of PROX1; P < 0.05.

PROX1 has been identified as an independent prognostic factor for survival in patients with World Health Organization grade II gliomas (22), In high−grade malignant astrocytic gliomas, PROX1 is highly expressed (15). Additionally, we screened publicly available clinical data of LowerGrade Glioma (LGG) from The Cancer Genome Atlas (TCGA; www.cancergenome.nih.gov), and found that PROX1 expression levels are significantly increased in glioblastoma tumors (n=516) as compared to healthy tissue (n=698) (**Figure 1A**). We also performed the Kaplan-Meier analysis in 516 Lower grade glioma patients from TCGA *via* the R software and revealed that high relative expression of PROX1 is associated with lower survival rates (**Figure 1B**). These observations are in agreement with the earlier studies that PROX1 acts as a tumor promoter in the pathogenesis of GBM (17).

### TPL depresses the expression of PROX1

**Figure 2:**
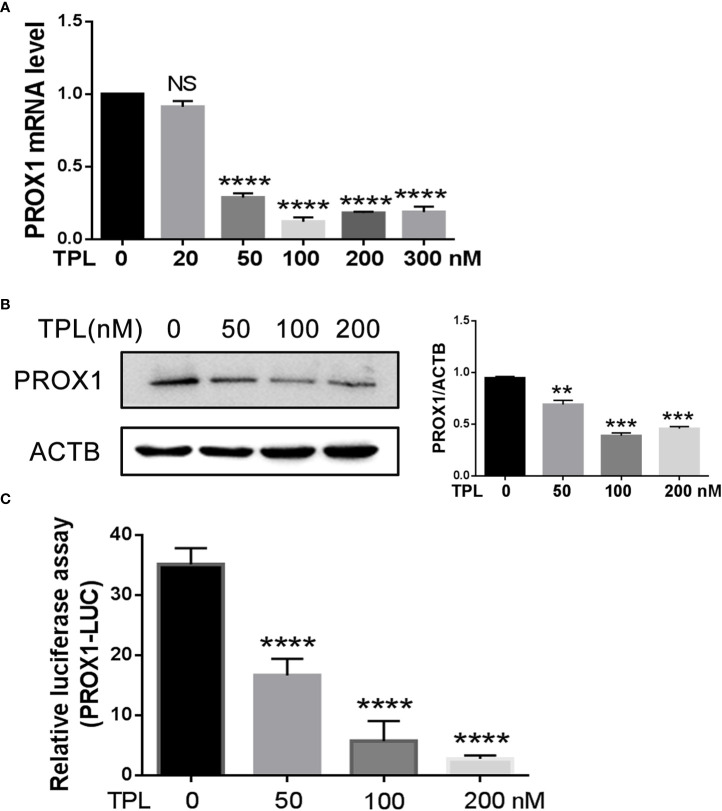
TPL transcriptionally depresses the PROX1 expression. **(A)** U251cells were cultured in 6 wells and treated with 0, 20, 50, 100, 200 or 300 nM TPL for24 h, and then cells were collected for QRT-PCR. ****, *p*<0.0001; NS, no significant. **(B)** U251cells were cultured in 6 wells and treated with 0, 50,100, 200 nM TPL for 24 h, and cells were lyzed with RIPA lysis buffer and used for Western blot with PROX1 antibody. **, *p*<0.01; ***, *p*<0.001. **(C)** The luciferase assay:U251cells were cultured in 24 wells and co-transfected with PROX1-LUC (0.25mg) and pRL-TK plasmid for 24 hours. Then cells were suffered to 0,50, 100 or 200 nM TPL treatments for 24 h. the cell were lyzed and the cell lysates were subjected to luciferase assay. ****, *p*<0.0001.

To initially address the effect of TPL on PROX1, we treated the U251 cells with different concentrations of TPL at 0, 50, 100, or 200 nM, and detected both the mRNA and protein levels of PROX1 using qRT-PCR and Western blot. As presented in **Figure 2A**, 50, 100, or 200 nM TPL treatments significantly reduced the mRNA levels of PROX1, but 20 nM of TPL had no obvious effect on the expression of PROX1. **Figure 2B** indicates that TPL markedly inhibited the protein levels of PROX1 in a dose-dependent manner. As reported previously, TPL Inhibits the global gene transcription in cancer cells by degradation the RNA polymerase II (Rpb1) (23). Thus, we hypothesized that TPL might transcriptionally depress the expression of PROX1. To confirm this hypothesis, we performed a dual-luciferase reporter assay in U251 cells using the PROX1 promoter plasmid (PROX1-LUC), which cloned ~ 3100bp of the 5’UTR sequence above the PROX1 transcription start site into the pGL3-basic vector. As shown in **Figure 2C**, 20, 50, or 100 nM TPL treatments obviously depress the luciferase activity of PROX1 promoter. These results indicate that TPL depresses the PROX1 expression.

### TPL inhibits the progression of GBM *via* PROX1

**Figure 3:**
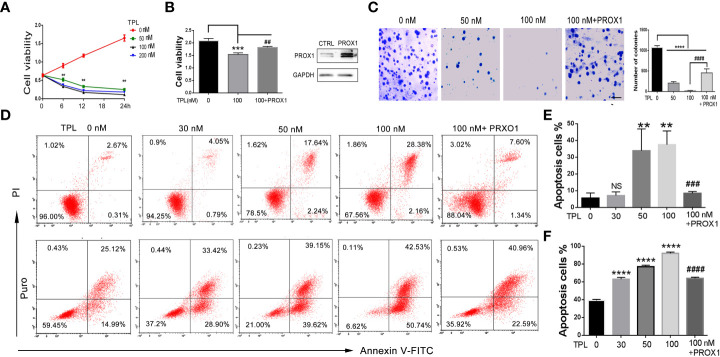
TPL depresses the glioma cell progression via PROX1. **(A)** CCK8 assay. U251 cells were cultured in 96 wells and treated with 0, 50, 100 or 200 nMTPL for 0, 6, 12, 18 and 24 hours, then cells were harvested to analyze the cell viability via CCK8 kit.**, *p*<0.01. **(B)** U251 cells were cultured in 96 wells and transfected with pcDNA3.1-PROX1 plasmid, and then treated with 100 nM TPL for 24 h. Over-expression of PROX1 partially rescues the inhibition of cell viability induced by TPL. Comparing with 0 nM, ***, *p*<0.001; Comparing with 100 nM, ##, *p*<0.01 **(C)** Soft agar assay. U251 cells were cultured in 96 wells and transfected with pcDNA3.1-PROX1 plasmid, and then treated with 0, 50 or 100 nM TPL for 24 h. The cells were then resuspended in a mixer with full media and matri-gel (1:1) and cultured for another 1-2 weeks. Comparing with 0 nM, ****, *p*<0.0001; Comparing with 100 nM, ##, *p*<0.01; Scale bar=10mm. **(D)** Apoptosis assay. U251 cells were cultured in 96 wells and transfected with pcDNA3.1-PROX1 plasmid, and then treated with 0, 30, 50 or 100 nM TPL (upper line) or TPL combined treatment with puromysin (5mg/ml) (lower line) for 24 h. The well treated cells were collected for apoptosis assay using via a flow cytometry. **(E)** Statistical analysis of apoptosis cells treated with TPL. Comparing with 0 nM, **, *p*<0.01; Comparing with 100 nM, ###, *p*<0.001, NS, no significant. **(F)** Statistical analysis of apoptosis cells treated with TPL and puromysin. Comparing with 0 nM, ****, *p*<0.0001; Comparing with 100 nM, ####, *p*<0.0001.

To address the role of TPL in Glioblastoma tumors, we detected the cell viability, apoptosis and growth rate using CCK8 assay, FITC-annexin V/PI staining, and soft agar colony formation assays, respectively. For the CCK8 assay, U251 cells were pre-cultured in 96-well plates and treated with TPL at 0, 50, 100, or 200 nM, then calculate the cell viability at 0, 6, 12, and 24h. As shown in **Figure 3A**, 50, 100, or 200 nM TPL treatments significantly inhibited the viability of the U251 cells in a dose-dependent manner, and its maximal effect was achieved with 100 nM. The soft agar colony assay showed a great degradation of colony-forming ability following 50 or 100 nM TPL treatments (**Figure 3C**). Furthermore, we did the apoptosis assay in U251 cells using TPL at 0, 20, 50, or 100 nM with or without puromycin (1.5μg/ml) for 24 h. As shown in **Figures 3D, E**, only 50, or 100 nM TPL treatments induced the apoptosis rate of U251 cells at 22% or 33%. However, when combined with puromycin, 30, 50, or 100 nM TPL treatments significantly increased the number of cells undergoing apoptosis at 63%, 79% or 93% (**Figures 3D, F**). Given the critical role of PROX1 in glioma progression and the inhibitory effect of TPL on PROX1 expression, it is possible that PROX1 play an essential role in TPL-mediated glioma inhibition. Then we transfected the U251 cells with PROX1 plasmid for 12 h, and treated with 100 nM TPL for another 24h, then cells were collected for CCK8, apoptosis and soft agar colony assays. And we found that PROX1 partially rescues cell viability (**Figure 3B**), the apoptosis (**Figure 3D**) and growth inhibition (**Figure 3C**) induced by TPL.

### TPL inhibits the proliferation of Glioblastoma

**Figure 4:**
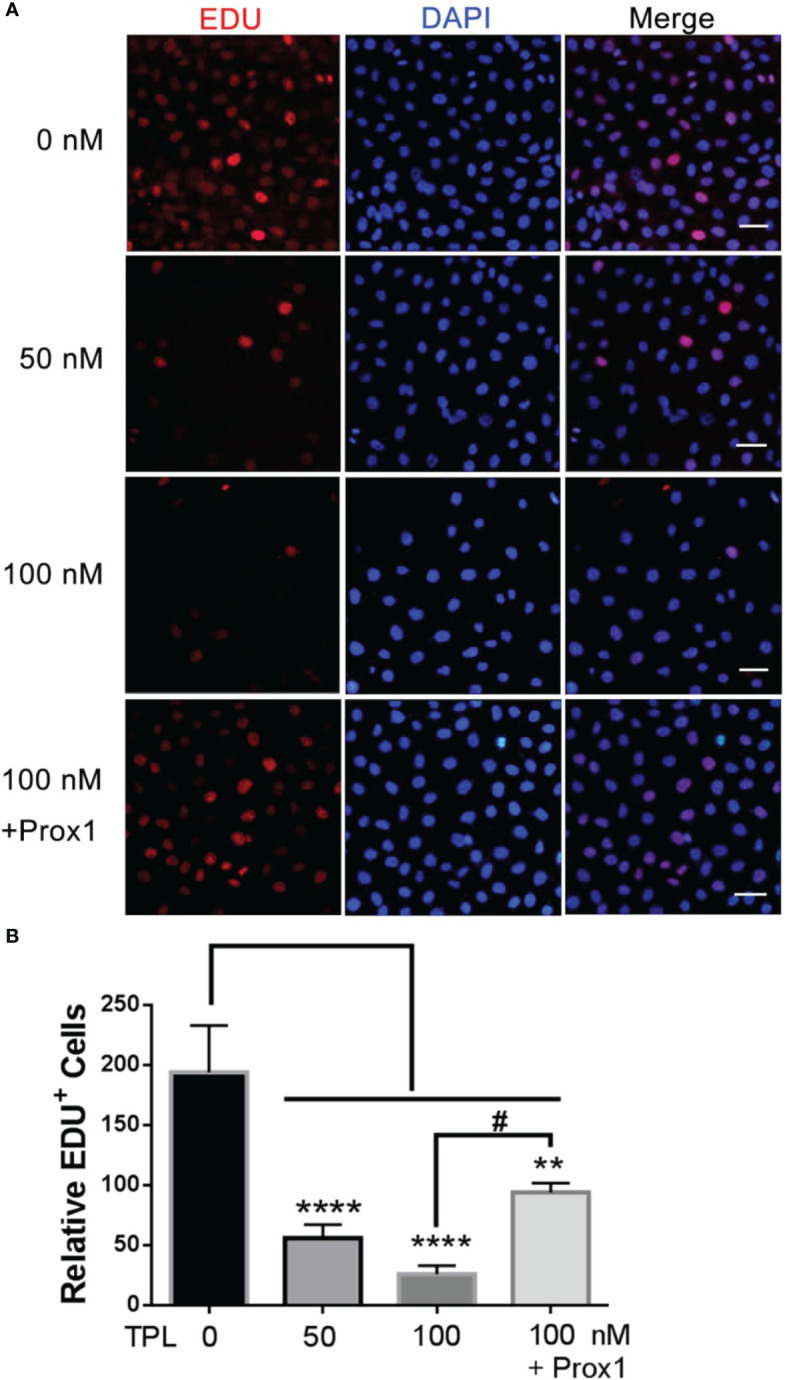
TPL inhibits the proliferation of glioma. **(A)** Cell proliferation assays.U251 cells were cultured in 24 wells and over-expressed PROX1, then treated with 0, 50, or 100 nM TPL. 12 hour later, 10 μM EDU was added and incubated for 12h, and cells were then used for EdU cell proliferation assays, which measure the incorporation of EdU into newly synthesized DNA. The nuclei were stained with DAPI. Scale bar=100μm. **(B)** Statistical analysis of relative EDU-positive cells. Comparing with 0 nM, **, *p*<0.01; ****, *p*<0.0001; Comparing with 100 nM, #, *p*<0.05.

**Figure 5:**
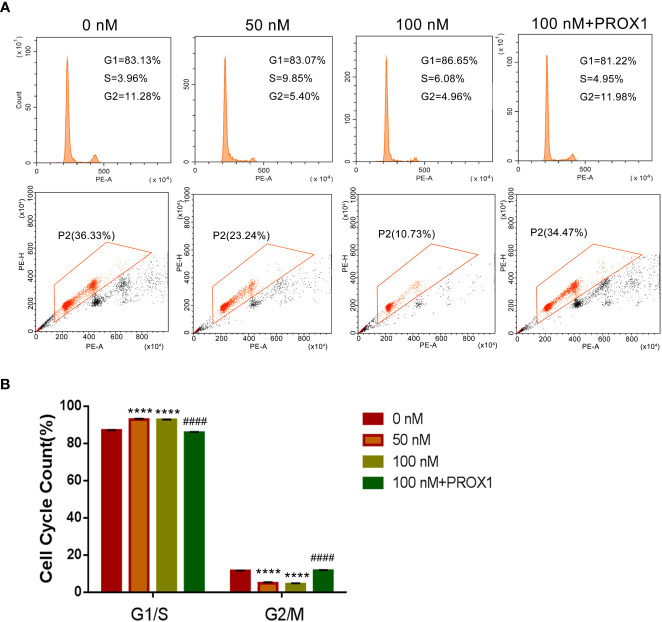
TPL arrests the cell cycle of glioma cells in G1/S phase. **(A)** Representative flow cytometry images of cell cycle assays using U251 cells were similar treated in **Figure 4A**. **(B)** Statistical analysis of the relative proportion of G1/S or G2/M in each cell-cycle phase. Comparing with 0 nM, ****, *p*<0.0001; Comparing with 100 nM, ####, *p*<0.0001.

To further confirm the effect of TPL on the growth of glioblastoma, we performed the cell cycle and proliferation assay in U251 cells, respectively. The cell cycle assay revealed that 50 or 100 nM TPL treatments obviously arrest the cell cycle at G1/S phase, and the over-expression of PROX1 could partially abolish the cell cycle arrest of TPL (**Figure 4**). In addition, the proliferation assay showed that 50 or 100 nM TPL treatments markedly inhibit the proliferation rate (EDU^+^ cells) of U251 cells, and PROX1 restore the proliferation inhibition induced by TPL (**Figure 5**).

### TPL inhibits the migration and invasion of GBM

**Figure 6:**
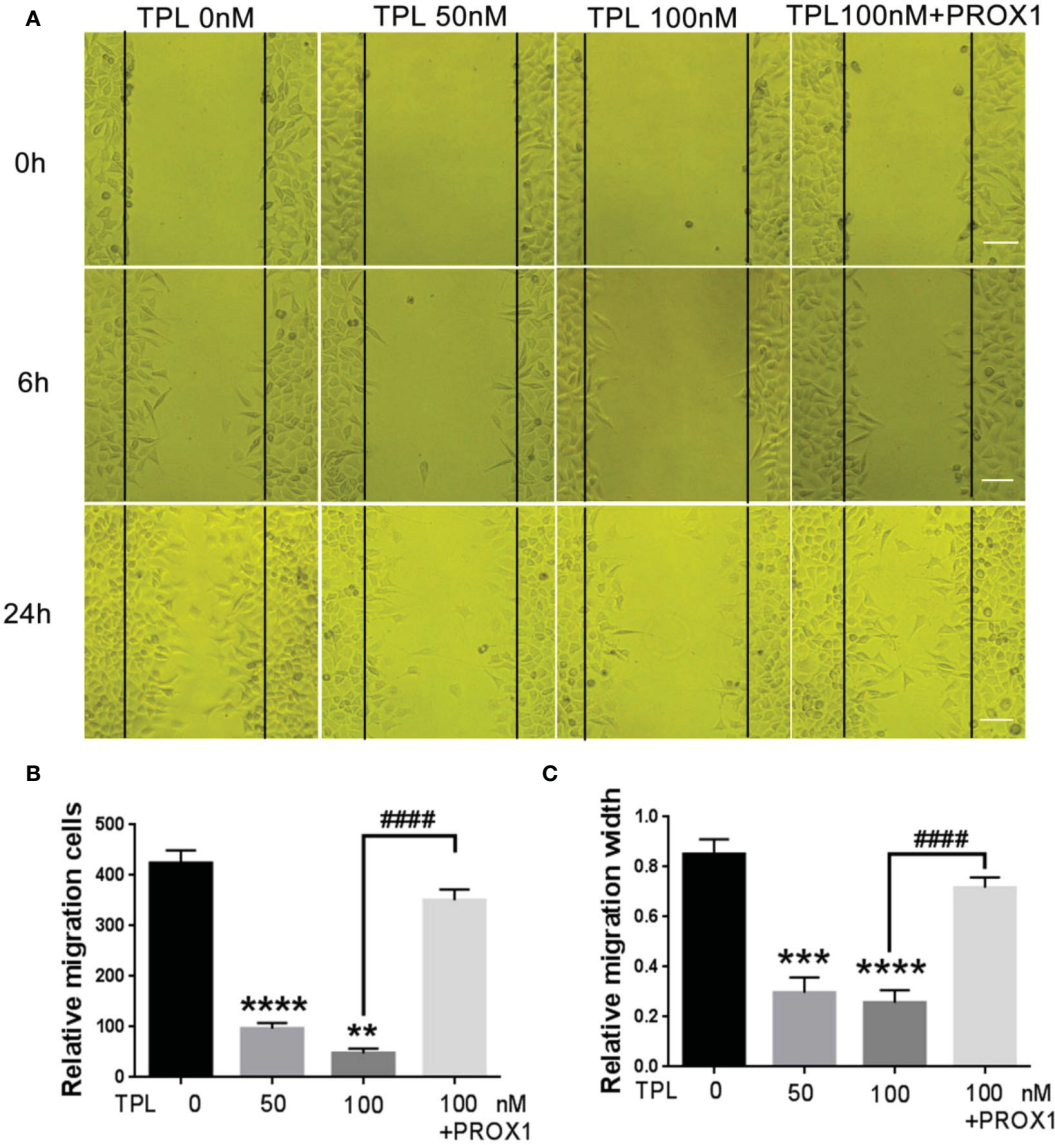
TPL inhibits the migration of glioma cells. **(A)** Representative cell migration images of wound healing assays using U251 cells were similar treated in **Figure 4A**. Scale bar=10mm. **(B, C)** Statistical analysis of the relative migration cells **(B)** and migration width **(C)** *via* Image J. Comparing with 0 nM, **, *p*<0.01; ***, *p*<0.001; ****, *p*<0.0001; Comparing with 100 nM, ####, *p*<0.0001.

**Figure 7:**
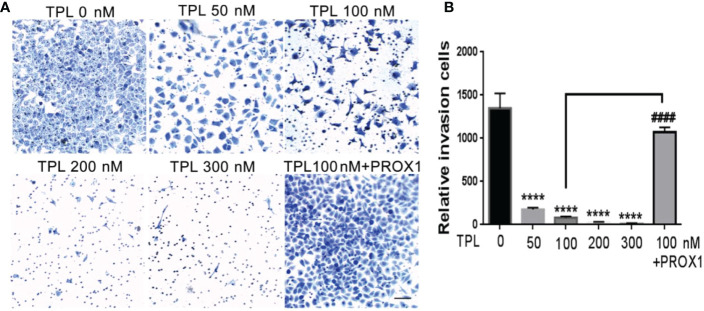
TPL inhibits the invasion of glioma cells. **(A)** Representative invasive images of transwell assays using U251 cells treated with 0, 50, 100, 200, 300 nM,or 100 nM TPL+ PROX1. Scale bar=10mm. **(B)** Statistical analysis of the relative invasion cells and revealed that TPL significantly inhibits the invasionof U251cells, but PROX1 partially blocks the inhibition. Comparing with 0 nM, ****, *p*<0.0001; Comparing with 100 nM, ####, *p*<0.0001.

Glioblastoma cells are highly aggressive, which is an important factor contributing to the poor prognosis of GBM (24). Therefore, we investigated the effect of TPL on the invasiveness of U251 cells. For migration assay, U251cells were cultured in 12 wells, and treated with TPL at 0, 50, 100 nM or 100 nM+PROX1 for 24 h, then cells were suffered to wound healing assay and graphed at 0, 6 and 24h. As shown in **Figure 6**, comparing to control group, 50 or 100 nM TPL significantly depress the migration cells (**Figures 6A, B**) and migration width (**Figures 6A, C**) of Glioblastoma cells. However, PROX1 enables the glioma cells to regain the migratory capacity. For transwell assay, the U251cells were prepared with TPL at 0, 50, 100, 200, 300nM or 100 nM+PROX1 for 24h, and cells were harvested, and re-cultured in Matri-gel coated chamber with 200 µL serum-free DMEM, while the lower part were added 500 µL DMEM containing 10% FBS and indicated concentration of TPL for 12h, then stained with crystal, washed with PBS and graphed. The results suggested that 50, 100, 200 or 300nM TPL dramatically reduce the invasive cells, and PROX1 make the U251 cells regain the invasive capacity (**Figures 7A, B**).

### TPL regulates the expression of the cell function-related genes

**Figure 8:**
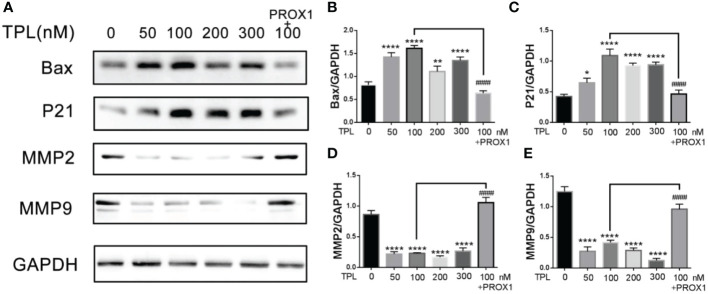
TPL regulates the expression of cell function-related genes. **(A)** U251 cells treated with 0, 50, 100, 200, 300 nM, or 100 nM TPL+ PROX1 for 24h, then the cells were lyzed and cell lysates were used for Western blot with indicated antibodies。**(B–E)** Statistical analysis of the relative protein level of CCND1, CCNE1, MMP9 and Bax, GAPDH acts as the internal control. Comparing with 0 nM, *, *p*<0.05; **, *p*<0.01; ****, *p*<0.0001; Comparing with 100 nM, ####, *p*<0.0001.

PROX1 was reported to promote the GBM progression *via* inducing the expression of matrix metallopeptidase 9(MMP9), which were the downstream targets of the NF−κB pathway (17). Our previous results have showed that TPL inhibits the cell cycle, proliferation, migration and invasion, and promote apoptosis of glioma cells. Thus, we detected the expression of the cell cycle related genes P21, and the apoptosis associated gene Bax, the invasive gene MMP2 and MMP9. As shown in **Figure 8**, 50, 100, 200 or 300 nm TPL significantly induced the expression of P21(**Figures 8A, C**), Bax (**Figures 8A, B**), and depressed the expression of MMP2 (**Figures 8A, D**) and MMP9 (**Figures 8A, E**). In addition, ectopic expression of PROX1 could rescue the effect of TPL on the expression of P21, Bax, MMP2 and MMP9 (**Figures 8A–E**). Those results suggest that TPL blocks the progression of glioma cells *via* targeting PROX1.

## Discussion

Glioblastoma remains a therapeutic challenge for researchers because of its rapid growth, extensive invasiveness, and poor prognosis after surgical resection, chemotherapy, and radiotherapy. TPL, a major component of the Chinese medicinal herb *Tripterygium wilfordii* Hook F, was reported to inhibit the growth of some tumors *in vivo* and *in vitro* (25). However, the mechanisms underlying the anti-tumor activity of TPL remain to be further explored. The present study revealed that TPL obviously inhibited the glioblastoma U251 cell growth, proliferation, migration, and invasion, but remarkably induced cell apoptosis. Mechanistically, the expression of PROX1 was significantly silenced after TPL treatments in a dose-dependent manner. And the cell cycle, apoptosis, and invasion-related genes regulated by PROX1 respond to TPL treatment. Moreover, over-expression of PROX1 distinctly reversed the TPL- induced inhibition of glioblastoma cell growth, migration, and invasion, as well as enhancement of apoptosis.

Inducing cancer cell apoptosis is considered to be the most effective method for cancer therapy (26). Previous studies have demonstrated the antitumor effects of TPL on glioblastoma (27). TPL was found to have significant inhibition of cell viability in Diffuse intrinsic pontine glioma (DIPG) patient-derived cell lines *in vitro* and *in vivo* (28). TPL was also reported to have selective cytotoxicity to patient-derived IDH1-mutated glioma cells (29). In this study, we also found that TPL inhibited the viability and growth rate of U251cells in a dose-dependent manner (**Figures 2A, C**). Moreover, TPL induced the U251cell apoptosis when the concentration of TPL exceeds 50nM (**Figures 2D, E**). More interestingly, when combined therapy with puromycin, just 30nM TPL significantly increased the number of cells apoptosis (**Figures 2D, F**). TPL possesses potent antitumor properties in various cancer cell types but is clinical application is limited due to its toxicity (5). For therapeutic purposes, it gives us a hint that when used in combination with other anti-tumor drugs, TPL can produce anti-tumor effects at a lower concentration with minimal toxicity to normal tissues.

Glioblastoma is characterized by rapid cellular proliferation, aggressive central nervous system invasion, and resistance to all known anticancer drugs (30). TPL have been reported to inhibit the proliferation and migration of numerous cancer cells, like colon cancer cells, medulloblastoma cells, hepatocellular carcinoma cells and neuroblastoma cells (6, 31–33). At present, the proliferation (**Figure 4**) and cell cycle (**Figure 5**) of the glioblastoma U251 cells was significantly blocked after TPL treatments. At the same time, the cell cycle inhibitor p21 was significantly increased (**Figures 8A, C**). Glioblastoma cells possess a high invasiveness potential, which contributing to poor prognosis (34). MMPs, especially MMP-2 and MMP-9, have been shown to be involved in cancer cell migration and invasion. In our study, the relative cell migration (**Figure 6**), invasion (**Figure 7**), and both the MMP2 (**Figures 8A, D**) and MMP9 expressions (**Figures 8A, E**) were decreased after 50 or 100 nM TPL treatments.

Depending on tumor types, PROX1 fulfills a dual role, acting as a tumor suppressor or tumor promoter. PROX1 is highly expressed and independently identified as the prognostic factor of grade II gliomas (22). From TCGA database, we found that the expression of PROX1 is also increased in LGG patients (**Figure 1A**), and its highly expression was associated with poor prognosis of survival (**Figure 1B**). These results suggest that PROX1 might have a pro-tumor effect on glioblastoma cells. This view was verified by Xu et al (17), and reported that PROX1 promoted the GBM cell growth, tumorigenesis, and invasiveness *via* activating p65 transcriptional activity through phosphorylation of IκBα. From this point of view, PROX1 can be used as a target of glioblastoma. Therefore, to determine the underlying mechanism of the anti-tumor activities of TPL, we examined the effect of TPL on PROX1. The QRT-PCR and Western blot results revealed that TPL depressed the mRNA (**Figure 2A**) and protein levels (**Figure 2B**) of PROX1 in a concentration dependent manner. And the luciferase assay showed that TPL blocked the PROX1 promoter activity (**Figure 2C**). These results suggested that TPL might transcriptionally depress PROX1 expression. Accompanying with the expression of PROX1, the effect of TPL on glioblastoma cell viability, proliferation, apoptosis, migration and invasion was partially attenuated, and on the expression of cell cycle, apoptosis and invasion–related genes were also weakened.

The present study has various limitations. Firstly, all the studies were performed in glioblastoma U251 cell *in vitro*, additional *in vivo* studies are required to confirm the antitumor impact of TPL on GBM. Secondly, how PROX1 regulates the expression of cell function-related genes P21, BAX, MMP2 and MMP9 remains to be fully elucidated. Finally, both TPL and PROX1 were reported to affect NF−κB activation, there is a lack of detection of NF−κB activation by PROX1 and TPL in glioblastoma cells.

Taken together, the present study verified that TPL has a tumor-suppressive effect against glioblastoma cells by depressing PROX1 expression. And we proposed that TPL could be an effective therapeutic medicine for glioblastoma treatment.

## Data availability statement

Publicly available datasets were analyzed in this study. This data can be found here: TCGA-LGG.

## Author contributions

CY performed the most experiment and collected and drafted the manuscript. YL Analyzed the data, SL, DL and MH assisted in some experiment. YQ and LL helped to revise the manuscript. WS designed the experiment and drafted the manuscript. XY designed the experiment and super-revised the manuscript. All authors contributed to the article and approved the submitted version.
